# Views of health service providers on obstacles to tuberculosis control in Ghana

**DOI:** 10.1186/2049-9957-2-9

**Published:** 2013-05-02

**Authors:** Joshua Amo-Adjei

**Affiliations:** 1 Department of Population and Health, University of Cape Coast, Cape Coast, Ghana

**Keywords:** Tuberculosis control, Barriers, Diagnosis and treatment, Health care providers

## Abstract

**Background:**

Although Ghana does not fall into the category of those countries which have a high burden of tuberculosis (TB), the disease does present considerable economic and health limitations to individuals infected with, and affected by, the disease, as well as to the health system in general. Despite this fact, insufficient studies have been done on the key barriers to controlling the disease. This paper presents results from an exploratory study on the constraints of controlling TB in Ghana based on the opinions of health service providers.

**Methods:**

In-depth interviews were conducted with frontline health workers involved in TB control in the country. Participants were purposively selected from a pool of national and regional, and district and facility level coordinators of the National Tuberculosis Control Programme (NTP). One key informant was also selected from an international non-governmental organisation (NGO) involved in TB-related activities in Ghana. Observations were utilised to complement the study. Data were analysed inductively.

**Results:**

Respondents identified the following as being constraints to TB control: clinical complication, bottlenecks in funding administration, quality of physical infrastructure, competition for attention and funding, unsatisfactory coordination between TB and HIV control programmes, a poor public-private partnership, and weak monitoring and evaluation of interventions.

**Conclusions:**

This paper provides evidence of some key barriers to TB control. The barriers, as reported, were generally health system-based. Although this list of barriers is not exhaustive, it would be useful to take them into account when planning for TB control, thus adopting a more rounded approach to TB management in the country. As well as that, further studies should be done to explore patients’ views on health service-related barriers to TB control.

## Multilingual abstracts

Please see Additional file 1 for translation of the abstract into the six official working languages of the United Nations.

## Background

Over the years, global disability-adjusted life-years (DALYs) reports have shown that communicable diseases contribute to about 24% of years of life lived with disability (YLD) and 19% of years of life lost (YLL) [1,2]. While some communicable diseases such as tuberculosis (TB) are curable, others such as the human immunodeficiency virus (HIV) are not. Yet out of the curable communicable diseases, TB is reported to be the most predominant cause of death globally due to high infectivity rates, poverty, malnutrition, and concurrent infection with HIV and acquired immune deficiency syndrome (AIDS) [1].

Despite the fact that drugs to treat and cure TB have existed for the past 50 years, significant challenges remain for TB prevention and control. Every 15 seconds, someone in the world dies from TB and infections that remain untreated have the potential to induce 10–15 other infections annually [3]. To meet the challenges of TB, the World Health Organization (WHO) declared TB as a public health emergency in 1993 [4], the only disease to get that description since WHO’s establishment in 1948.

Although a number of interventions have been rolled out by both international institutions and local governments to fight TB, efforts are sometimes plagued by challenges. These challenges include, but are not limited to, clinical, socio-cultural/economic and institutional factors. Clinically, for instance, concurrent infection of TB and HIV remains to be one of the main constraints to effective TB control. In fact, available evidence shows that HIV infection reactivates latent TB infection. This accelerates new infections and re-infections, leading to a lifetime risk ranging from 10-20% among individuals having dormant TB. It is also one of the most common causes of morbidity and mortality among people living with HIV [5,6].

Socio-culturally, some popular constraints to TB control include default of, and non-compliance to, efficient treatment therapies, which is often heightened by physical distance [7]. Others include illiteracy and inadequate knowledge of TB, poor patient–provider interaction and perceived side effects of TB drugs [8]. As well as that, unemployment [9]; lack of, or inadequate, social support [10]; marijuana use and alcohol consumption during treatment are some other barriers to treatment adherence [11].

At the institutional or healthcare level, factors such as insufficient funding resulting in a decrease in screening coverage, low salaries, staff shortages, irregularities in drug supplies and out-dated infrastructure are issues worth considering. Institutional impediments may affect responsiveness of health systems to the needs of patients [12].

Over the last two decades or so, TB control in Ghana has seen some improvements in terms of case notification and treatment success. Treatment success was around 44% in 1997, rising to 88% in 2010 [13]. In spite of the existence of a large volume of literature on the constraints to TB diagnosis and treatment, Dimitrivo et al. [12] pointed out that context is relevant for studies of this nature. The authors argued that barriers may vary within different contexts, and it is therefore important that studies from various contexts are encouraged to help identify possible divergences and convergences.

The rising incidence of TB, which was partly due to the rising incidence of HIV/AIDS, in the early 1990s called for an establishment of a formal programme for TB control [13]. Formalised TB control under the auspices of the National TB Control Programme (NTP) has been in existence since 1994. The WHO-recommended treatment regimes of directly observed treatment, short-course (DOTS) combined with passive surveillance were instrumental at the initial stages. With the introduction of the TB Enablers Package, supervised treatment is provided in both communities and health facilities. The Enablers Package is an arrangement where patients, health service providers and community members (who serve as treatment supporters) are given a stipend to encourage treatment completion. Under the community care intervention, patients, in collaboration with health workers, identify the supporters who are to observe TB patients swallow their drugs. Although these strategies, in concert with others, have resulted in positive impacts on TB control, there are still obstacles to case detection and treatment outcomes. For instance, the case detection rate in 2008 was about 27%, less than the 75% minimum detection performance set by the WHO [13].

This article draws on qualitative data to explore health system-based barriers to effective diagnosis and treatment of TB in Ghana. While this study is specific to Ghana, it may also provide insights into some potential barriers to effective diagnosis and treatment of TB in Sub-Saharan Africa in general.

## Methods

This study was part of a larger study that was intended to explore tuberculosis (TB) control policies in Ghana. Other issues covered in the study include the public-private partnership, integration of TB and HIV, political commitment, as well as barriers to TB control from the perspective of health service providers.

The study followed a qualitative approach, drawing key informants from staff of the NTP at the national and sub-national (regional, district and health facility) levels. The study relied on a qualitative or interpretivist paradigm with the understanding that this provides a better way of tapping into real-life experiences of health workers with respect to what they consider to be observed barriers. These categories of staff are considered to be the appropriate stakeholders as they are directly in charge of developing and implementing policies of the NTP. Participants were selected purposively and, in all, 24 respondents were selected and distributed as follows: three national officers of the NTP, four regional coordinators, four district coordinators, eight facility coordinators and four respondents from private facilities. One respondent, who is not a Ghanaian but from an international, non-governmental organisation (NGO) involved in TB-related activities in the country, was also selected. In each facility, the most senior staff was selected because they were considered to have the most experience in TB control. In most DOTS clinics, there was only one nurse but if there was more than one, the most senior nurse was selected. None of the targeted participants declined to participate in the study – this could have been because the NTP formally introduced the study to all potential respondents and in this way showed its support to the respondents.

The sample for the study was arrived at on the basis of saturation [14]. While there is no theoretical benchmark for arriving at saturation, it has been suggested that saturation could be achieved with five to 30 interviewees [15-17] and Guest et al. [18] posits that it is possible that a sample of six interviews 'may [be] sufficient to enable development of meaningful themes and useful interpretations' (2006:78). In this study, therefore, the responses were deemed saturated when no new themes were emerging.

Respondents were chosen from four regions which are known to have high TB cases in the country. These were the Greater Accra, the Eastern Region, Ashanti and the Western regions. At the national level, three national officers from the NTP were selected to provide more insights into policy design and implementation of a public-private partnership at all stages of the control programme. In each region, the TB coordinator for each district reporting the highest cases in that region was chosen for an in-depth interview, and one public and one private facility were selected from each of the districts. At each health facility, the most senior person in charge of TB activities was selected for the key informant interview.

Within group (intra-method) triangulation method was employed for the data collection. In other words, two qualitative data collection techniques, interviews and observations were combined to generate data for the study. Respondents were asked to indicate what they believed to be the constraints to TB control in the country. This formed the list of the specific barriers discussed in this article. Respondents were probed for additional information of how exactly these obstacles affect TB control programmes. Consent was obtained from individual interviewees by explaining the purpose of the study. All respondents gave verbal consent, as well as consenting to be tape recorded. The duration of the interviews ranged from 30 to 60 minutes. Data collection and transcription were done concurrently between March 2012 and June 2012 by the author.

Observations were made in two ways. The first observation was made at all study sites concurrently with the interviews being conducted. This provided an opportunity to observe conditions at various clinics and hospitals as part of the validation steps in qualitative research. The second observation was in the form of the author participating in a one-day regional forum of district TB coordinators organised by the NTP. The author was not introduced as a researcher and his presence was therefore unlikely to affect discussion. The forum provided an opportunity to observe proceedings from an outsider's perspective and to take notes on some of the pertinent issues discussed. Among some of these issues were the management of the relationships between disease control officers and clinicians, formats for reporting TB cases, new trends in diagnosis and the Global Fund to Fight HIV, Tuberculosis and Malaria (GF) funding conditions.

The Institutional Review Board of the University of Cape Coast gave ethical approval for the study. Individual participation in the study was voluntary and no incentives were provided for participation. Perhaps due to the NTP's support for the study, none of the targeted participants opted out from it. Despite this, it is unlikely that the support from the NTP introduced biases into the study as a quality control measure of complementing the individual interviews with observation was undertaken.

Since this study was mainly exploratory, the data analysis was informed by inductive coding [19]. By this approach, analytic categories were not predetermined and the specific themes were allowed to emerge from the data itself. Transcripts of the interviews were repeatedly read to aid in the identification of themes. Two experts in qualitative research, who are colleagues of the author, crosschecked the codes identified for consistency. The draft report was given to three randomly selected research participants to vet the draft as a means of respondent validation. Ambiguities identified through both peer review and respondent validation were resolved in an iterative manner [18-20]. Thick descriptions [20] were also used to improve the validity of the findings.

## Results

The following emerged from the respondents as being some of the key barriers that constrained tuberculosis (TB) control activities. These include clinical complications, bottlenecks in funding administration, physical infrastructure, unsatisfactory coordination between TB and HIV control programmes, a poor public-private partnership, and weak monitoring and evaluation of programmes and interventions.

### Clinical complications

For the most part, the development of fixed-dose combination (FDC) for treatment has been instrumental in reducing default rates among patients. However, there were specific apprehensions about the size of the FDC tablet. A number of DOTS centre nurses observed that certain patients complained about the size of the dose, which they feared could be counterproductive and result in defaulting. One nurse observed:

Some patients often complain about the size of the doses they take, think that the drugs are too big and have a host of other complaints. Others face a big blow when they are not cured after six months and are required to take injections for another two months. One reason for this is the cost of commuting to facilities on a regular basis for injections (DOTS Centre Nurse, Region 3, Private Facility).

Added to this were perceptions of how some physicians viewed TB management. Some participants claimed that a section of clinical staff were not interested in TB and that perhaps this low interest could be arising from how TB is managed. A shift from a purely clinical management of the disease, which has been the way in the past, to a mix of social and clinical treatment was identified.

… There is low clinician involvement due to the structured nature of the programme. Usually, when clinicians notice a TB case, they immediately refer the patient to a TB coordinator. The problem I see with this attitude is that it is possible that a patient does not only have TB; there could be other conditions involved as well which would require a clinician's attention. Assuming a patient has malaria or is malnourished, or has any other conditions that can affect TB management, clinicians' services will be required as TB caregivers are mandated to provide TB treatment only (Coordinator, Region 2).

According to some respondents, the low involvement of clinicians in TB control has resulted in low detection of possible TB cases among clinicians. In many instances, clinicians agreed to non-participation and often quickly referred TB suspects to disease control officers, justifying their actions based on the perception that activities of the TB control programme are heavily centred on disease control officers. One clinician (who was first trained as a disease control officer and, later, a medical doctor) indicated that some disease control personnel in one district resisted him when he showed an interest in TB. According to him, their opposition was borne out of ‘fear’ of him potentially taking over what was their ‘preserve’. The following details his experience:

In my first meeting with staff in the directorate as a district director of health services, when I indicated to the staff that I was going to assume responsibilities as district TB coordinator, they looked at me in awe. They could not accept how a clinician would want to be a TB coordinator … TB control is being treated like Yaws control because disease control officers kept everything, including the funds, drugs, to themselves because of 'nokofio' [money] (Clinician and District Director of GHS, Region 4).

A respondent indicated that the seemingly poor relationship between disease control staff and clinicians/physicians perhaps arises from the financial packages involved in TB control. Under current arrangements, TB patients, their treatment supporters and health workers are given tokens such as a transportation allowance, mobile phone credit and food as a means of motivating them to stick with the treatment programme. One respondent felt that if the disease control staff were also the spending officers, this could increase clinicians' interest in TB-related activities. He noted: 'the Enablers Package is what is bringing the confusion' (Director of Clinical Services, Region 4).

These disagreements between clinicians and public health staff could fit into the ‘medicalised’ model of health delivery in which clinicians perceive health delivery as their preserve. Consequently, attempts to include others are treated with opposition. As one respondent revealed, the integrated primary healthcare approach, which has been adopted from the beginning of the NTP, was not fully accepted by some clinicians and this opposition, according to some respondents, still prevails in some cases. Ayee [21] argued that in public policy change, there are 'demons' that can oppose policies through overt and covert strategies, and these demons may be at play in this scenario.

### Bottlenecks in funding administration

Research participants expressed varied opinions about how they are able to access funds. Generally, these stemmed more from frustrations rather than from commendation, and varied among respondents. Issues mentioned mostly dealt with delays in accessing funds for various activities. Whereas the national programme receives funds directly from donors and the Government of Ghana (GoG), this is not the case at the regional, district and facility levels where funds do not go directly to the TB control coordinators. Consequently, experiences varied from place to place and some respondents rightly conceded to internal issues that may be peculiar to their institution. One respondent's view was:

Although funds do come from the Canadian International Development Association, the Global Fund and others, releasing funds for activities is beset with significant challenges. We go through hell before funds are released to us… I suspect our hospital administration is intentionally delaying the release but why this is the case, I cannot really tell. What I do know, however, is that all other hospitals/clinics in this district receive their share of funds to use on patients and for other petty expenses before we get ours… The accounting processes are also not practical. For instance, those who are supposed to release the funds demand receipts for even sachet water bought for patients. While I acknowledge that proper financial records should be kept, there should be allocations for general miscellaneous items rather than having to account for every little thing… I wish funds for TB programmes were sent directly to the programme officer at the various hospitals to reduce the bottlenecks involved in making funds available (DOTS Centre Nurse, Region 3, District A, Public Hospital).

At the regional TB review forum, which the author participated in as an observer, some sub-national personnel involved in TB control were implicated as being 'good spenders' of money, but sometimes fared poorly in accountability. As a result, some regional directors of health were hesitant in releasing funds to district coordinators because of their poor reporting systems. This was also borne out of the strict financial accountability to GF. Some regional and district directors felt that it would be in their personal and institutional interests to withhold funds and instead leave the responsibility of those funds to the NTP to avoid being drawn into any sort of financial malfeasance. Such fears could be borne out of the recent implications of misappropriating GF grants – in one Sub-Saharan African country, certain TB control programme officers have been imprisoned for their inability to adequately account for grants provided by the GF. In this larger context, the anxieties characterising the release of funds to certain coordinators may be justified.

### Competition for attention and funding

In an integrated health system, as is the case in Ghana, health workers are not primarily employed to be fixed to one unit or department, except in the case of specialists, such as anaesthetists. During discussions with respondents about barriers to TB control, it emerged that various activities compete for health workers' attention and time, making them unable to devote all their attention towards TB-related activities. One recurrent area was national immunisation days (NIDs). Respondents indicated that during NIDs, all activities come to a halt, partly due to integration. Below is one participant's view:

TB competes with other diseases for attention. Within Ghana Health Service (GHS), there is that kind of competition for diseases to be seen on the agenda. For instance, during NIDs, every programme, regardless of its relevance, will have to wait because NIDs are seen as being more nationally inclined (National Officer 2).

However, competition does not permeate into funding at the local level. At the international level where there is competition, it occurs among countries rather than among diseases. This could be because several disease control programmes have specific organisations or donors on their agenda. For example, the Danish government through Danish International Development Agency (DANIDA) is seen as being more TB friendly. The GF, which is biased towards TB, HIV/AIDS and malaria, has dedicated budgets for the respective diseases. Due to this, respondents did not perceive competition for donor funds as being a barrier to TB control.

### Physical infrastructure

By mid-2012, there were 1,057 DOTS and 254 diagnostic centres distributed across 170 districts of Ghana. From the respondents’ perspectives, the ratio of these physical infrastructures to population is acceptable. Quantitatively, the NTP's performance is so far acceptable. A regional coordinator made this reservation:

We don’t have many problems with [physical] infrastructure, such as labs. It’s even been speculated that, presently, we don’t need any more labs (Coordinator, Region 4).

It is the quality of this infrastructure that was the most recurrent concern of frontline personnel. The author's personal observations about the centres are consistent with the participants’ expressions:

Infrastructure is bad, ventilation in this centre is bad, [and] we share this place with the injection and dressing room. Our drug storage facility, as you can see, is very poor. There is no restroom in this centre (DOTS Centre, Region 3, District A, Public Hospital).

There was also disquiet about the location of DOTS centres in certain hospitals – both public and private facilities expressed this. From the data, two issues in respect to location were derived. The first was that some centres were too close to maternal and child health (MCH) centres, which could subject mothers and their children to avoidable infections. In some areas, it was observed that the DOTS centres were next to MCH centres and health workers were very concerned about this:

… Look at this small room; it is the same room that we use for HIV counselling and testing. It is not the best. Ideally, the clinic should be a bit further from the main wards due to the infectious nature of tuberculosis. This will prevent patients/patrons of other health services from contracting the disease. In this facility, for instance, the MCH centre is our next neighbour and you can just imagine the risk children and their mothers are exposed to (DOTS Centre Nurse, Region 3, Private Facility).

The other issue about location related to privacy. Selected participants at DOTS centres noted that locations do not promote privacy. According to some respondents, there were occasions when patients refused to come to the DOTS centres for supervised treatment when they saw people they knew in, and around, the clinics accessing other services, such as injury dressing. The respondents perceived such behaviour as emanating from the stigma associated with the disease. One respondent remarked:

There is also no privacy in this facility – we share this place with the injury dressing room and this does not bode well for patients' privacy. For instance, when some patients approach the facility and notice their acquaintances around, they retreat and hide until their acquaintances leave. Sometimes when you [the health worker] are lucky, they [the patients] will send a bystander to come and pick up the drugs for them. They do this in fear of being stigmatised by their relatives, friends or co-tenants … (DOTS Centre Nurse, Region 3, Public Hospital).

Although, field observations were consistent with the views of the respondents, in other health facilities, the NTP, in collaboration with hospital managers, had provided well-ventilated DOTS clinics where patients’ privacy could somehow be maintained.

### A poor public-private partnership

According to some respondents, certain private facilities had high expectations about the tangible benefits they stood to gain from the partnership. For instance, some private facilities had the impression that their physical infrastructure would undergo significant improvements as part of the partnership. Once their part of the bargain had been delivered through the provision of services to TB patients, the NTP was supposed to fulfil its part. A respondent surmised:

I feel that we could not manage expectations of private facilities very well. Expectations of private facilities were high, as many private providers thought their involvement in tuberculosis control was going to result in massive infrastructural changes, as well as other benefits to their facilities. When those expectations were not met, most of them sat back. Initially, they were supported with funds and other logistics, but with the funds dwindling, some have recoiled (Regional coordinator, Region 2).

Whereas facilities were improved in some cases, in the majority of private facilities such packages could not be provided. Therefore, some of the private providers who felt side lined have withdrawn service provision to TB patients.

### Unsatisfactory coordination between TB and HIV control programmes

For purposes of effectiveness and efficiency, some countries, for example, Cambodia [22], are pulling TB and HIV resources together in a seamlessly integrated manner. This is even more relevant given the diminishing funds being provided for TB and HIV programmes. To that extent, even minimal coordination between programmes (sharing of information on co-trimoxazole utilisation, for instance) is expected to achieve a positive balance between TB and HIV management. Unfortunately, this desirable expectation does not seem to be prevailing in the country, according to the respondents. For instance:

While we [the NTP] make sure to screen almost all (about 98%) of our patients for HIV and give them [the National AIDS Control Programme (NACP)] the returns, they hardly supply us with their returns. We have tried several times to streamline things, but their usual complaint is about their workload and lack of time to do the paperwork. We requested tuberculosis data from their [NACP] side and the response was that they don’t have an appropriate system for capturing this data. We felt this could be a reasonable excuse so we helped them develop a data-capturing system. We were then expecting returns from them at the end of this quarter. Unfortunately, when I did a follow-up, they had misplaced the form and I had to give them another. So you see where the problem is coming from? (Regional coordinator, Region 3)

### Weak monitoring and evaluation

One of the key foundations of successful TB control is the quality of monitoring and evaluation (M&E). Within the current Global Fund (GF) to Fight HIV, Tuberculosis and Malaria funding conditions, strong M&E are tied to about 15% of funding that should be made available to countries in Round 10 of the GF call for proposals.

I think we can do better for it to become vibrant and rigorous. It is through monitoring and supervision that we can collect the correct data needed for a smooth programme. Occasionally, you review data from the sub-national level and you realise that the data is a bit different from the level below or above. However, through comprehensive monitoring and supervision, such inconsistencies can be corrected (National Respondent 3).

Concerns about weak M&E was re-echoed at almost all levels of the NTP structure – some regional coordinators felt that M&E at the national level was inadequate. Similarly, district level personnel felt that regional coordinators were not proactive in M&E. Overall, there appears to be a hierarchical weakness associated with M&E in Ghana’s NTP. A regional coordinator, however, attributed the weaknesses in M&E to delays in receiving funds from the NTP in Ghana. She observed:

The first quarter of the year (2012) has ended and we are yet to receive funds for routine activities. So you can just imagine the kind of situation we find ourselves in (Regional coordinator, Region 1).

Under the present M&E situation, there is fear that Ghana may not be able to access the 15% of additional funding that would have been made available to the NTP if its M&E processes were efficient. The difficulty here is whether effective M&E precedes adequate funding or the reserve. According to some of the respondents, it would seem reasonable that adequate and timely provision of funds would provide the impetus for tightening M&E procedures. Declining the NTP further funding because of their M&E practices when initial ones have not been effectively utilised would then provide objective basis for applying sanctions.

## Discussion

This study explored the perceptions of stakeholders for tuberculosis (TB) control in Ghana. Generally, the views the respondents expressed are substantially tilted towards health-system barriers rather than patient barriers. The only perceived barrier associated with patients related to their attitudes towards intake of a fixed-dose therapy. The remaining barriers were about frictions between public health and clinical staff, bottlenecks in the release of funding, the quality of infrastructure, slack attitudes towards TB/HIV programmes, a bridled public-private partnership, and weak monitoring and evaluation practices.

Respondents admitted to inadequacies existing in funding programmes, however, respondents, especially at the facility level, are aware of the funding gaps that exist in meeting health needs in certain developing countries and are coping with the situation. They did, however, express grave concerns about delays and bureaucracies associated with the release of funding for routine activities [23]. For instance, the TB Enablers Package, which is supposed to be provided to patients at the point of care, sometimes has unduly delays. This creates obstacles to TB activities, such as home verification and patient support funding.

There were also complaints about the quality of rooms allocated for TB treatment. This appears to be one of the complex barriers to TB treatment. The stigma attached to TB makes it prudent for a fair balance in properly locating TB clinics. Whereas segregation of DOTS centres may heighten stigma among the general public and healthcare providers not directly involved in TB diagnosis and treatment [24], situating them close to other vulnerable healthcare users, such as pregnant women, mothers and children, may also expose them to avoidable risks. In addition, it was reported that poor ventilation in some of the clinics further worsened such location issues. In a larger context, the location of DOTS centres sometimes raises ethical issues that feed the stigmatisation. A historical analysis of TB stigmatisation revealed that the persistence of the stigma could partly be attributed to a segregation of patients (e.g., [23]). Indeed, in a study in urban Takoradi, Ghana, some community members, both TB infected and uninfected, unanimously agreed that the segregation of TB wards coupled with ‘excessive’ use of protective gloves by health personnel when attending to TB patients provides the basis for stigmatising TB patients in non-health workers. Due to such concerns, isolation of TB wards is not presently being encouraged although the risk of nosocomial infection could be high [24].

Nevertheless, as the respondents indicated, the key problem related to the quality of the infrastructure. The concerns regarding the quality of infrastructure have also been highlighted in past studies. For instance, Birx et al. [25] observed that one major challenge to the triad of TB, HIV and malaria control programmes is infrastructural difficulties, in both quality and quantity. Given the high infectivity of TB, it is important to address both quality and quantity of infrastructure concurrently, instead of the present situation where the emphasis seems to be on quantity.

The perceived frosty relationship between clinicians and public health staff over diagnosis and treatment of TB may be related to the highly-medicalised (professionally and politically structured) nature of the health system in Ghana. Based on the current structure, medical doctors are placed above any other staff in the system. While this is not sufficiently injurious to the health delivery system, there are perceptions of competition between clinicians and public health workers as far as TB is concerned, particularly about who should manage the funding. Clinicians have somehow become apathetic towards TB diagnosis and treatment, leaving it to the management of public health professionals who are not trained to deal with treatment complications.

Although useful for TB treatment and diagnosis, the public-private partnership implementation was perceived to have been hasty, without setting clear modalities on the expectations and obligations of the partners. The expectations of the private sector seem to be about tangible benefits such as rehabilitation of laboratories. With some of these expectations not met, certain private facilities withdrew their services. Porter and Kramer [26] argued that partnerships are bound to fail when mutual goals are not created for the sake of the partnership. This seems to be the case with the present situation with respect to the private sector involvement in TB control. Both the public and the private sector are somehow operating with different values. The WHO [27] observed beneficence, non-maleficence, autonomy and equity as being essential ingredients for successful public-private partnerships. Unfortunately, these ingredients appear to be missing in the current public-private partnership in TB control in Ghana.

The study also found signals of weak coordination and collaboration between TB and HIV programmes, with some respondents considering this to be an indication of barrier to diagnosis. The WHO policy on collaborative TB/HIV programmes [28] calls on NTPs and NACPs to work towards a minimum of facility-level integration. However, the present situation in Ghana is far from this ‘ideal’: DOTS and antiretroviral therapy (ART) clinics in some parts of the country are located separately. In certain places, either one of the facilities exists without the other. This increases the cost of treatment among co-infected patients. For instance, as a result of separate locations of DOTS and ART clinics, the cost of commuting between clinics could be high and that becomes an obstacle to compliance to treatment. Underlying this barrier is a tacit reluctance of both TB and HIV control programmes to forge and implement proposals of the Working Group on TB and HIV. At best, the Working Group appears to exist in name only. However, Ansah et al. have found positive impacts in having facility-level integration of TB and HIV diagnosis and treatment services [29].

The study had some limitations. Studies based on perceptions such as this are likely to suffer from exaggeration or underestimation of issues, and this has taken into account when interpreting the findings. Also, this study does not provide representative views on barriers to TB control in Ghana, but only focuses on the experiences of respondents in the study areas. These experiences are, therefore, prone to differ from site to site. Future studies may employ quantitative techniques, which are able to quantify the externalities of the barriers to TB control highlighted in this paper. Such studies will obviously enhance the understanding of the specific barriers that need urgent attention and consequent presentation to policy makers.

## Conclusion

In this paper, an attempt has been made to explore some fundamental barriers to tuberculosis control in Ghana, drawing on the experiences of health workers directly involved in routine management of the disease. The barriers, as reported by the respondents, are generally health system-based. In order to address these barriers, it is recommended that the NTP adopts a more holistic approach to deal with them.

## Competing interests

The author declares no competing interest.

## Supplementary Material

Additional file 1 Multilingual abstracts in the six official working languages of the United Nations. Click here for file
