# Supplementary material for: Views of health service providers on obstacles to tuberculosis control in Ghana
Source: Infect Dis Poverty. 2013 May 2;2:9. doi: 10.1186/2049-9957-2-9 (PMC3710189; doi:10.1186/2049-9957-2-9)
Supplement: Additional file 1 — Multilingual abstracts in the six official working languages of the United Nations. [file 2049-9957-2-9-S1.pdf]

Translation of the abstract into the six official working languages of the United Nations

وجهات نظر مقدمي الخدمات الصحية حول العقبات أمام مكافحة السل في غانا

جوشوا أمو-أدجي

ملخص

معلومات أساسية: على الرغم من أن غانا لا تقع ضمن فئة البلدان التي تعاني من ارتفاع عبء مرض السل، فإن هذا المرض لا يفرض قيودًا اقتصادية وصحية كبيرة على الأفراد المصابين به، والمتضررين منه، وكذلك على النظام الصحي بشكل عام. وعلى الرغم من هذا، فقد أُجريت دراسات غير كافية حول أهم العوائق في مكافحة ذلك المرض. وتعرض هذه الورقة نتائج دراسة استكشافية حول معوقات مكافحة مرض السل في غانا استنادًا إلى آراء مقدمي الخدمات الصحية.

الطرق: أُجريت مقابلات مستفيضة مع العاملين في الخطوط الأمامية في مجال الصحة من المشاركين في مكافحة السل في البلاد. وتم اختيار المشاركين بشكل مقصود من بين مجموعة من منسقي البرنامج الوطني لمكافحة السل على المستوى الوطني والإقليمي وعلى مستوى المناطق والمنشآت. وقد تم اختيار مرشد أساسي أيضًا من منظمة دولية غير حكومية مشاركة في الأنشطة المتعلقة بمرض السل في غانا. واستُخدمت أيضًا الملاحظات المسجلة لأغراض الدراسة. وقد تم تحليل البيانات عن طريق الاستقراء.

النتائج: حدد المجيبون المعوقات أمام جهود مكافحة السل فيما يلي: التعقيدات الطبية، ومأزق إدارة الأموال، وجودة البنية الأساسية المادية، والتنسيق غير المرضي بين برامج مكافحة مرض السل وفيروس نقص المناعة البشرية (الإيدز)، والشراكات الضعيفة بين القطاعين العام والخاص، فضلاً عن ضعف رصد وتقييم التدخلات.

الاستنتاجات: تقدم هذه الورقة أدلة على بعض المعوقات الرئيسية لمكافحة السل. كانت هذه المعوقات، كما ذكرت، قائمة سواءً على أساس النظام الصحي وعلى أساس المرضى. وعلى الرغم من أن قائمة المعوقات هذه ليست شاملة، فسيكون من المفيد وضعها في الاعتبار عند التخطيط لمكافحة السل، وبالتالي اعتماد منهج أكثر تخصيصًا لإدارة السل في البلاد. فضلاً عن ذلك، ينبغي إجراء المزيد من الدراسات لاستكشاف وجهات نظر المرضى حول المعوقات المتعلقة بالخدمات الصحية لمكافحة السل.

Translated from English version into Arabic by Muhammad Assayed, through

TRANSLATORS
WITHOUT BORDERS

加纳的卫生服务提供者对防治结核病工作中的困难的看法

Joshua Amo-Adjei

摘要

引言：虽然加纳并未进入结核病高负担国家的行列，但是该病确实对加纳的经济和感染者的健康造成巨大影响，并影响到卫生系统。尽管如此，并未对该病防治工作的主要困难进行过足够的研究。本文在加纳卫生服务提供者的意见基础上对结核病防治工作的制约因素进行了探索性研究。

方法：对参与加纳结核病防治工作的一线卫生工作者进行了深入调查。在全国结核病防治项目中挑选国家级、地区级、区级和基层协调员作为调查对象。一个重要的调查对象来自在加纳的一个与结核病相关的国际非政府组织。也使用了相关的观察内容。对数据进行归纳分析。

结果：受访者确定了以下几个结核病防治工作的制约因素：临床并发症、基金管理的瓶颈、基础设施的质量、结核病和艾滋病防治规划间协调欠佳、公共和私营部门间的伙伴关系不良、监测薄弱和干预措施评估不力。

结论：本文发现了一些结核病防治工作中的主要问题。正如所报道的一样，这些问题均以卫生体系和患者为基础。尽管上述所列的制约因素不够详尽，但是在制定结核病防治规划时，考虑这些因素，对在国家结核病管理工作中采取更加适合的方法还是有用的。下一步应对结核病防治工作中与卫生服务相关的患者意见进行深入研究。

Translated from English version into Chinese by Yang Pin, through

CDC CHINESE CENTER FOR DISEASE CONTROL AND PREVENTION
NATIONAL INSTITUTE FOR PARASITIC DISEASES

Point de vue des fournisseurs de services de santé concernant les obstacles liés au contrôle de la tuberculose au Ghana

Joshua Amo-Adjei

Extrait

Contexte : Bien que le Ghana n'entre pas dans la catégorie des pays fortement touchés par la tuberculose, la maladie entraîne des limites importantes en matière d'économie et de santé pour les personnes infectées et pour les personnes concernées, ainsi que pour le système de santé en général. Malgré cela, trop peu d'études ont été réalisées au sujet des principaux obstacles qui empêchent de contrôler la maladie. Cet article présente les résultats d'une étude préliminaire portant sur les contraintes du contrôle de la tuberculose au Ghana, fondée sur l'avis des fournisseurs de services de santé.

Méthodes : Des entrevues poussées ont été réalisées auprès de travailleurs de la santé sur le terrain qui participent au contrôle de la tuberculose dans le pays. Les participants ont été délibérément sélectionnés dans un bassin de coordinateurs à l'échelle nationale et régionale, à l'échelle du quartier et à l'échelle de l'installation dans le cadre du programme national de contrôle de la tuberculose. Un informateur principal a également été sélectionné au sein d'une organisation non gouvernementale (ONG) internationale participant aux activités liées à la tuberculose au Ghana. On a également utilisé des observations pour l'étude. Les données ont été analysées de manière inductive.

Constatations : Les personnes interrogées ont indiqué que les éléments suivants nuisaient au contrôle de la tuberculose : complications d'ordre clinique, goulots d'étranglement dans l'administration des fonds, qualité de l'infrastructure physique, mauvaise coordination entre les programmes de contrôle de la tuberculose et du VIH, faibles partenariats entre les secteurs public et privé et contrôle et évaluation insuffisants des interventions.

Conclusions : Le présent article apporte la preuve de l'existence de certains obstacles essentiels au contrôle de la tuberculose. Comme cela a été signalé, les obstacles concernent à la fois le système de santé et les patients. Bien que cette liste d'obstacles ne soit pas exhaustive, il serait utile d'en tenir compte au moment de la planification du contrôle de la tuberculose, ce qui permettrait d'adopter une approche plus complète de la gestion de la maladie dans le pays. En outre, il faudrait mener des études supplémentaires pour examiner le point de vue des patients concernant les obstacles au contrôle de la tuberculose liés aux services de santé.

Translated from English version into French by Mélanie Bernard, through

TRANSLATORS
WITHOUT BORDERS

Взгляд работников здравоохранения на проблемы в борьбе с туберкулезом в Гане.

Джошуа Амо-Аджей

Реферат

Общая информация: Хотя Гана не относится к категории стран, в которых туберкулез (ТБ) широко распространен, болезнь налагает значительные экономические и медицинские ограничения на инфицированных и пострадавших от заболевания лиц, а также на систему здравоохранения в целом. Несмотря на это, исследований основных проблем в борьбе с заболеванием проведено недостаточно. В данной статье представлены результаты поискового исследования о проблемах в борьбе с туберкулезом в Гане, полученные на основании опроса мнений работников здравоохранения.

Методы: С медицинскими работниками, непосредственно участвующими в борьбе с туберкулезом в стране, были проведены глубинные интервью. Участников целенаправленно отобрали среди национальных, региональных и районных координаторов Национальной программы борьбы с туберкулезом, а также среди координаторов отдельных учреждений. Кроме того, один ключевой респондент являлся сотрудником международной неправительственной организации (НПО), участвующей в деятельности по борьбе с туберкулезом в Гане. В исследовании также использовали результаты наблюдений. Данные анализировали индуктивно.

Результаты: Респонденты определили следующие проблемы в борьбе с туберкулезом: клинические осложнения, недостатки в управлении фондами, качество физической инфраструктуры, неудовлетворительная координация между программами контроля ТБ и ВИЧ, недостаточное государственно-частное партнерство, а также слабые мониторинг и оценка проводимых мероприятий.

Выводы: В этой статье перечислены некоторые основные проблемы в борьбе с туберкулезом. Обозначенные проблемы существуют как на уровне системы здравоохранения, так и на уровне пациентов. Хотя представленный перечень проблем не является исчерпывающим, их было бы полезно учитывать при планировании мероприятий по борьбе с туберкулезом, приняв тем самым более сбалансированный подход к обеспечению контроля ТБ в стране. Помимо этого необходимо провести дальнейшие исследования, чтобы изучить мнения пациентов о проблемах в борьбе с ТБ, связанных с медицинским обслуживанием.

Translated from English version into French by Stanislav Bakunov, through

TRANSLATORS
WITHOUT BORDERS

Los puntos de vista del personal médico sobre los obstáculos en el control de la tuberculosis en Ghana

Joshua Amo-Adjei

Síntesis

Antecedente: Aunque Ghana no entra dentro de aquellos países que sufren de una gran carga de casos de tuberculosis (TB), la enfermedad sí presenta considerables limitaciones económicas y de salud a los individuos infectados, y afectados, por la enfermedad y el sistema de salud en general. A pesar de esto, se ha estudiado poco sobre las dificultades claves en el control de la enfermedad. Este artículo muestra los resultados de un estudio exploratorio sobre las restricciones en el control de la tuberculosis en Ghana fundamentándose en las opiniones del personal médico.

Métodos: Se llevaron a cabo entrevistas detalladas con los trabajadores de la salud involucrados en primera línea en el control de la tuberculosis en el país. Los participantes fueron deliberadamente seleccionados de un grupo de coordinadores a nivel nacional, regional, de distrito y de instalaciones del Programa Nacional de Control de la Tuberculosis. Un informante clave también fue seleccionado de una organización internacional no gubernamental (ONG) involucrada en actividades relacionadas con la tuberculosis en Ghana. También se hicieron observaciones en el estudio. Los datos se analizaron inductivamente.

Hallazgos: Los encuestados identificaron lo siguiente como barreras en el control de la tuberculosis: complicaciones clínicas, atascos en la administración de fondos, calidad de las infraestructuras físicas, coordinación insatisfactoria entre los programas de control de TB y VIH, la mediocridad de las colaboraciones entre el sector público y el privado, y débiles supervisiones y evaluaciones de intervenciones.

Conclusiones: Este artículo ofrece evidencia de algunos impedimentos en el control de la tuberculosis. Como se ha relatado, éstas son del sistema de salud y de los pacientes. Aunque esta lista de dificultades no es exhaustiva, sería de utilidad considerarla cuando se planifique el control de la tuberculosis, y adoptar así un acercamiento más completo en la gestión de TB en el país. Además de eso, se deberían hacer más estudios para explorar los puntos de vista de los pacientes sobre las trabas relacionadas con los servicios de salud en el control de la tuberculosis.

Translated from English version into Spanish by Eva Castilla, through

TRANSLATORS
WITHOUT BORDERS
